# High expression of GFAT1 predicts poor prognosis in patients with pancreatic cancer

**DOI:** 10.1038/srep39044

**Published:** 2016-12-20

**Authors:** Caiting Yang, Peike Peng, Lili Li, Miaomiao Shao, Junjie Zhao, Lan Wang, Fangfang Duan, Shushu Song, Hao Wu, Jie Zhang, Ran Zhao, Dongwei Jia, Mingming Zhang, Weicheng Wu, Can Li, Yefei Rong, Lei Zhang, Yuanyuan Ruan, Jianxin Gu

**Affiliations:** 1 Department of Biochemistry and Molecular Biology, School of Basic Medical Sciences, Fudan University, Shanghai 200032, P.R. China; 2 Key Laboratory of Glycoconjugate Research Ministry of Public Health, School of Basic Medical Sciences, Fudan University, Shanghai 200032, P.R. China; 3 Department of General Surgery, Zhongshan Hospital, Fudan University, Shanghai 200032, P.R. China; 4 Institute of Biomedical Science, Fudan University, Shanghai 200032, P.R. China

## Abstract

Pancreatic cancer is one of the most lethal of all types of cancer, with the 5-year survival rate ranging only at 6–7%. The aberrant glucose metabolism is one of the hallmarks of cancer cells, and as a branch of glucose metabolism, hexosamine biosynthesis pathway (HBP) has been reported to play a critical role in the insulin resistance and progression of cancer. Glutamine:fructose-6-phosphate amidotransferase (GFAT1) is the rate-limiting enzyme of the HBP; nevertheless, the prognostic value of GFAT1 in pancreatic cancer remains elusive. In this study, we found that the expression of GFAT1 was increased in pancreatic cancer samples compared to peri-tumor tissues. High expression of GFAT1 was positively associated with lymph node metastasis, pTNM stage and shorter overall survival (OS) in pancreatic cancer patients. GFAT1 was identified as an independent prognosticator for OS, and combining GFAT1 expression with pTNM stage generated a predictive nomogram, which showed better prognostic efficiency for OS in patients with pancreatic cancer. In summary, high GFAT1 expression is identified as an independent predictor of adverse clinical outcome in our small number of pancreatic cancer patients, and the practical prognostic nomogram model may help clinicians in decision making and the design of clinical studies.

To date, pancreatic cancer has a high mortality rate and is the 7^th^ most frequent cause of cancer-related death^1^. Since most pancreatic cancer patients keep asymptomatic until it worsens, they are often diagnosed at an advanced stage when the 5-year survival rate ranges only at 6–7%^2^. Even for early-stage pancreatic cancer, the median survival of patients following resection is only 24–25 months in the setting of adjuvant or neoadjuvant chemotherapy^3^. The high rate of invasion and metastasis represents the major cause for its poor prognosis. Metastasis to distant organs, such as the liver, peritoneum, lungs and the bones, is commonly found when diagnosed, and makes surgical resection impossible for the patients. Besides, the nature that pancreatic cancer can spread along the nerves also attributes to its poor prognosis^4^. Traditional tumor-node-metastasis (TNM) classification systems could provide a predictive model for patients, but they still have limited capacity to determine different outcomes when referring to the asymptomatic nature in early stage and limitations of current detection technologies of pancreatic cancer. Therefore, it is still particularly urgent to establish a better prediction model and seek a prognostic biomarker which features high sensitivity, specificity and accuracy.

Deregulated glucose uptake and metabolism have been well recognized as a common feature of cancer cells^5^,^6^. Unlike most normal cells, many transformed cells derive a substantial amount of their energy from aerobic glycolysis, converting glucose to lactate rather than metabolizing it in the mitochondria through oxidative phosphorylation^5^,^6^. As a branch of glucose metabolism, 2–5% of glucose is channeled into the HBP and isomerized in two enzymatic steps to yield fructose-6-phosphate^7^. GFAT1 then transfers the amide group from glutamine to fructose-6-phosphate to generate GlcN-6-P in the first and rate-limiting step of HBP^8^. Moreover, pancreatic cancer cells displays addiction to glutamine and are sensitive to glutamine starvation^9^. So GFAT1, a glutamine-requiring enzyme, integrates both glucose and glutamine metabolism and may play an important role in pancreatic cancer progression. The dysregulation of GFAT1 has been found in breast cancer and is reported to be associated with tumor progression and relapse^10^. A previous study also indicates a possible correlation between GFAT1 gene variation and pancreatic cancer risk^11^. However, the protein level and clinical significance of GFAT1 expression in pancreatic cancer remains unclear.

In this study, we used immunohistochemistry (IHC) approach to detect the expression of GFAT1 in pancreatic cancer, and assessed its associations with clinicopathologic features and prognosis. In addition, we explored whether incorporation of pTNM stage and GFAT1 expression could establish a model for better predicting the outcome of patients with pancreatic cancer.

## Results

### GFAT1 is overexpressed in pancreatic cancer

**Figure 1:**
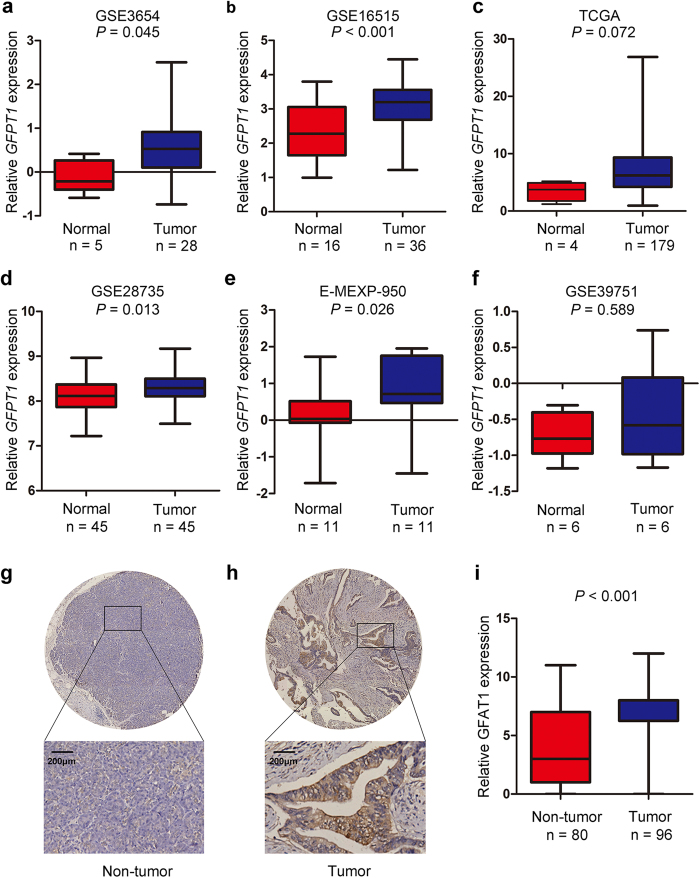
The expression patterns of GFAT1 in pancreatic cancer tissues. **(a–f)** Relative expression of GFAT1 mRNA in pancreatic cancer and normal pancreatic tissues in GSE3654 **(a)**, GSE16515 **(b)**, TCGA datasets **(c)**, GSE28735 **(d)**, E-MEXP-950 **(e)** and GSE39751 **(f)**. **(g,h)** Representative IHC staining images of GFAT1 and its regional magnification in pancreatic cancer tissues and non-tumor tissues. Scale bar = 200 μm. **(i)** IHC score of GFAT1 expression in pancreatic cancer tissues and non-tumor tissues.

To understand whether GFAT1 was involved in pancreatic carcinogenesis, we first examined the mRNA expression patterns of GFAT1 in pancreatic cancer tissues from reported GEO, ArrayExpress and TCGA datasets. We found that the GFAT1 mRNA expression was increased in tumor tissues in GSE3654 (*P* = 0.045), GSE16515 (*P* < 0.001), GSE28735 (*P* = 0.013) and E-MEXP-950 (*P* = 0.026) datasets (Fig. 1a,b,d,e), while no statistically significant increment of GFAT1 mRNA levels was observed in the tumor tissues from TCGA and GSE39751 dataset (Fig. 1c,f).

We next investigated the protein expression of GFAT1 in pancreatic cancer samples and adjacent non-tumor tissues. Immunohistochemical (IHC) assay revealed that the protein expression of GFAT1 was up-regulated in pancreatic cancer samples compared to peri-tumor tissues (*P* < 0.001) (Fig. 1g–i). The staining of GFAT1 was highly heterogeneous in tumor cells, including both the staining intensity and staining frequency (Supplementary Tables 1–3). Moreover, among the different cellular compartments of the tumor tissues, GFAT1 was strongly stained in the epithelial tumor cells, and relatively low expression of GFAT1 was detected in the islets (Supplementary Fig. S1a,c). No or faint staining of GFAT1 was found in stromal area and acinar cells (Supplementary Fig. S1b,d).

We also have analyzed the mRNA expression of another two hexosoamine pathway components, phosphoacetylglucosamine mutase (PGM3) and UDP-N-acetylglucosamine pyrophosphorylase (UAP1). PGM3 mRNA levels were found to be down-regulated in pancreatic cancer in the GSE28735 dataset, while no significant changes were observed in the other five datasets (Supplementary Fig. S2). UAP1 mRNA expression was also not altered in most datasets, while opposite changes was observed in the GSE28735 and E-MEXP-950 datasets (Supplementary Fig. S3).

### Correlations between GFAT1 expression and clinicopathological features in pancreatic cancer patients

To further evaluate the protein level of GFAT1 in pancreatic tumor tissues, we detected the expression of GFAT1 with immunohistochemical staining analysis and determined the correlations between GFAT1 expression and clinicopathological features in 96 pancreatic cancer samples. According to the results conducted by receiver operating characteristic (ROC) curve analysis, IHC score of 6 was determined as the cut-off to dichotomize the patients into GFAT1 low group (score, 0–6; n = 24) and GFAT1 high group (score, 7–12; n = 72) (Supplementary Fig. S4). The association between GFAT1 expression and clinicopathological variables in pancreatic cancer patients was analyzed with the chi-square test, and the result was listed in Table 1. Among the variables, patients with higher expression of GFAT1 significantly tended to be diagnosed at advanced pTNM stage (*P* = 0.033) and have higher rates of lymph node metastasis (*P* = 0.020). No other clinicopathologic variables showed a significant correlation with GFAT1 expression.

### Correlations between GFAT1 expression and overall survival in pancreatic cancer patients

**Figure 2:**
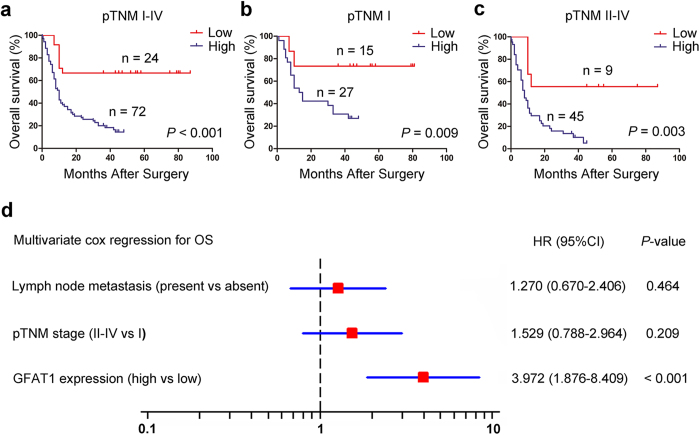
The predictive value of GFAT1 expression in patients with pancreatic cancer. **(a–c)** Kaplan-Meier survival analysis showing the relationship between GFAT1 expression and overall survival in all patients **(a)**, patients at pTNM I stage **(b)** and patients at pTNM II -IV stage **(c)**. **(d)** Cox multivariate analysis identified the independent prognostic factors for overall survival for patients with pancreatic cancer.

We next explored the relationship between GFAT1 expression and overall survival using Kaplan-Meier analysis. The results demonstrated that GFAT1 expression was negatively associated with overall survival of pancreatic cancer patients (*P* < 0.001) (Fig. 2a). To further evaluate the efficiency of GFAT1 expression in stratifying patients with different pTNM stages, we divided the patients into early (I) and advanced (II–IV) groups. In both the pTNM I and pTNM II +IV subgroups, GFAT1 expression showed statistically significant value in predicting the outcomes of pancreatic cancer patients (Fig. 2b,c). These data suggest that GFAT1 expression is correlated with overall survival for patients with pancreatic cancer.

### GFAT1 expression is identified as an independent prognosticator in patients with pancreatic cancer

We also conducted univariate Cox analysis to identify the prognostic significance of clinicopathological factors for overall survival. Lymph node metastasis (*P* = 0.004), pTNM stage (*P* = 0.002), and GFAT1 expression (*P* < 0.001) were found to be risk factors for survival in patients with pancreatic cancer (Table 2). Further adjustment of covariate factors using multivariate Cox analysis identified GFAT1 expression (*P* < 0.001) as the independent risk factor for pancreatic cancer (Fig. 2d). These data indicate that high expression of GFAT1 is an independent factor that predicts poor prognosis in patients with pancreatic cancer.

### Combination of GFAT1 expression with pTNM stage generates a better predictive model for overall survival of pancreatic cancer patients

**Figure 3:**
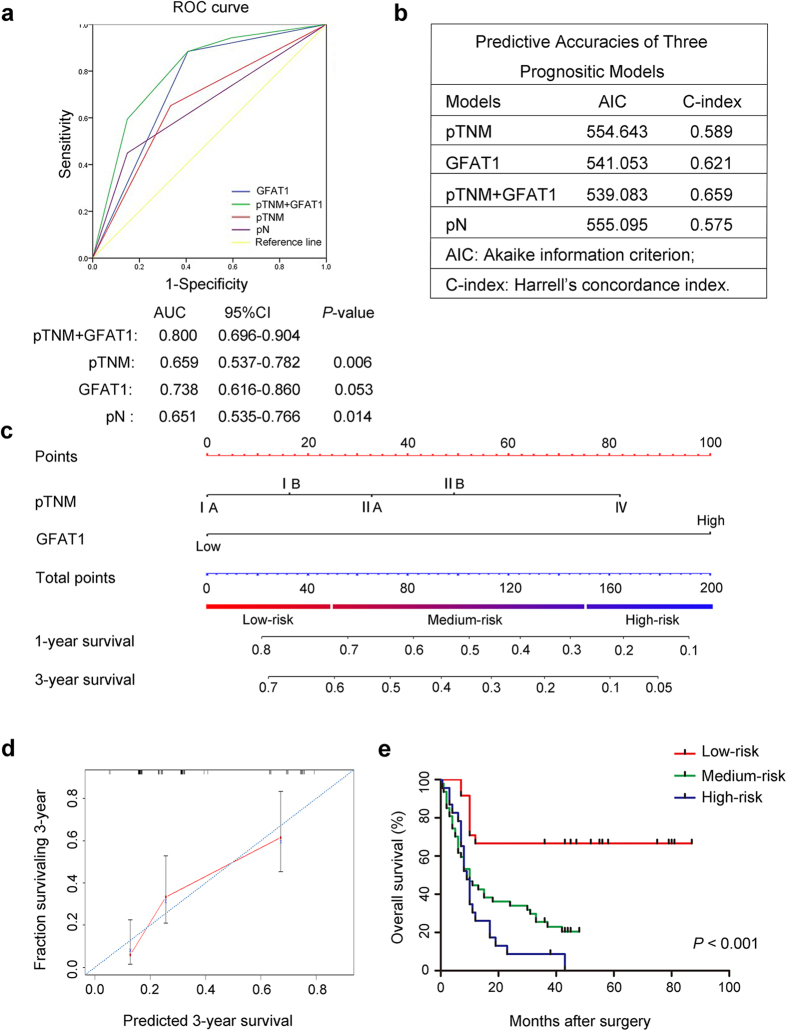
Combination of GFAT1 expression with pTNM stage generates a better predictive model for overall survival of pancreatic cancer patients (n = 96). (**a**) ROC curve analysis of the sensitivity and specificity of the predictive value of the pTNM stage model, GFAT1 model, the combined model and lymph node metastasis (pN) model. (**b**) AIC and Harrell’s C-index analysis of the comparison of the predictive accuracies of pTNM staging, pN and GFAT1 expression. (**c**) Nomogram for predicting clinical outcomes integrated GFAT1 expression (Low/High) with pTNM classification (IA, IB, IIA, IIB, IV). In the nomogram, higher total point predicts worse prognosis. Addition of pTNM classification (0 for “IA”, 16 for “IB”, 33 for “IIA”, 49 for “IIB” or 82 for “IV”) and GFAT1 expression (0 for “Low” or 100 for “High”) for each patient correspondingly gives the total point. (**d**) Calibration plot for nomogram predicted and observed 3-year survival rate. Calibration curves for nomogram predicted 3-year overall survival performed well with the ideal model. Line of dashes: ideal model; vertical bars, 95% confident interval. (**e**) Kaplan–Meier curves of overall survival based on risk score calculated by nomogram. *P*-value was assessed by log-rank test.

To establish a more sensitive model for predicting the outcomes of patients with pancreatic cancer, we combined GFAT1 expression and pTNM stage to create a prognostic score system. ROC curve analysis showed that the predictive value of GFAT1 alone (AUC [95% CI], 0.738 [0.616–0.860]) was higher than that of pTNM stage (AUC [95% CI], 0.659 [0.537–0.782]). The combination of GFAT1 and pTNM stage also revealed better prognostic value (AUC [95% CI], 0.800 [0.696–0.904]) than pTNM stage alone with statistical significance (Fig. 3a). In addition, the Harrell’s concordance index (C-index) for the combination of pTNM stage and GFAT1 was 0.659, higher than that for pTNM stage (0.589); the Akaike information criterion (AIC) was 554.64 when estimated according to pTNM stage alone, and it decreased to 539.08 when estimated in combination with GFAT1 expression (Fig. 3b). These results suggest that incorporation of GFAT1 expression into pTNM stage could establish a better predictive model for the overall survival of pancreatic cancer patients.

Based on the results of ROC analysis, we further constructed a nomogram model that integrated pTNM classification with GFAT1 expression for better stratifying patients with different prognosis. In this nomogram, a higher total point predicted a worse prognosis. The total point was raised by adding the score of the pTNM classification (0 for “IA”, 16 for “IB”, 33 for “IIA”, 49 for “IIB” or 82 for “IV”) and GFAT1 expression (0 for “Low” or 100 for “High”) for each patient (Fig. 3c). The calibration curve for predicting 3-year overall survival shows that the nomogram performs well with the ideal prediction model (Fig. 3d). Based on the risk score, patients were stratified into three subgroups, including subgroups I for low risk score (<25%), subgroup II for medium risk score (25%-75%) and subgroup III for high risk score (>75%). Kaplan-Meier analysis revealed that scoring with the nomogram effectively discriminated the risk of postoperative survival in pancreatic patients (Fig. 3e, *P* < 0.001).

## Discussion

Pancreatic cancer is currently one of the deadliest cancers with poor prognosis. Traditional predictive models for patients have limited capacity to determine different outcomes of pancreatic cancer. Carbohydrate antigen19–9 (CA 19–9) has been considered as a prognostic factor for pancreatic cancer, whereas the prognostic value of CA19–9 is still controversial^12^,^13^. Other pathologic prognostic factors, such as TNM classification systems, tumor size and lymph node (LN) metastasis, ignores the underlying molecular and cellular processes during the carcinogenesis of pancreatic cancer. Therefore, the construction of more effective prognostic system for clinical practice of pancreatic cancer is urgent. Our results demonstrate that expression of GFAT1 is increased in pancreatic cancer and is associated with poor prognosis of patients. In addition, GFAT1 could create a better predictive model for the outcomes of pancreatic cancer patients in the combination with pTNM stage. Therefore, the level of GFAT1 expression is a potential prognostic marker which might provide more approaches for predicting the outcome of pancreatic cancer.

The role of GFAT1 in cancer has also drawn more attention these years. A recent study demonstrates that high expression of GFAT1 predicts worse progression and worse pathologic outcomes in breast cancer^14^. In addition, it is reported that overexpression of GFAT1 induces the expression of mesenchymal marker in lung adenocarcinoma cells, suggesting that GFAT1 stimulates the EMT process^15^. In accordance with these findings, recent data indicate that GFAT1 inhibitors are found to be effective in cancer treatment^16^,^17^. Some studies showed the efficacy of glutamine analogs, the inhibitors of GFAT1, in inducing significant tumor regressions in cancer cells^17^,^18^,^19^,^20^ as well as in various human xenograft tumors (colon, mammary or lung) transplanted in athymic mice^21^. Moreover, on account of the success of these glutamine analogs *in vitro*, the inhibitors of GFAT1 were found to be effective in reducing tumor burden in soft tissue sarcoma, bone sarcoma, mesothelioma and colorectal carcinoma patients^22^,^23^. Therefore, targeting GFAT1 may provide new adjuvant approaches for the treatment of pancreatic cancer in clinical practice.

The end product of the HBP, UDP-GlcNAc, is substrate for oligo- and polysaccharide synthesis^24^. Therefore, the dysregulation of GFAT1 expression is tightly associated with glycosylation alteration in cancer cells. It has been well recognized that changes in traditional N- and O-linked glycosylation is correlated with oncogenesis^25^. In addition, UDP-GlcNAc is also used as the substrate for *O*-linked β-N-actylglucosamine modification (*O*-GlcNAcylation) on nucleocytoplasmic and mitochondrial proteins^26^. Hyper-*O*-GlcNAcylation was found in multiple types of human malignancies, and considered as a general feature of cancer cells. Numerous evidences indicate that *O*-GlcNAcylation is involved in the regulation of epigenetics, metabolism, transcription, translation, protein stability and signaling transduction, and is critical for tumor progression^27^. Hence, the increased expression of GFAT1 may contribute to the aberrant glycosylation pattern in pancreatic cancer cells.

Dense composition of stromal cells and extracellular matrix (ECM) composed largely of collagen is a common feature of PDAC. It has been reported that transforming growth factor-β1 (TGF-β1) is one of the strongest inducers of ECM production during fibrogenesis and desmoplasia within PDAC^28^. Interestingly, studies have demonstrated that GFAT1 could promote TGF-β1 expression in mesangial cells and fibroblasts^29^,^30^. Therefore, up-regulation of GFAT1 in tumor cells may not only be involved in pancreatic carcinogenesis, but also contribute to the ECM deposition through enhancing TGF-β1 expression.

Though our data suggest that the expression of GFAT1 is increased in pancreatic cancer, we found the differences in GFAT1 mRNA levels comparing normal and tumor tissues are relatively small (Fig. 1a,b,d,e) or not significant (Fig. 1c,f) in the reported datasets. Nevertheless, significant changes in the GFAT1 protein expression were apparently observed in our cohort (Fig. 1g–i). One possible reason is due to the small sample size of these microarray datasets. Another explanation is that GFAT1 is up-regulated in pancreatic cancer at post-transcriptional level rather than transcriptional level. Indeed, previous research suggests that GFAT1 could be controlled at post-translational level since it has a relatively short half-life of 1 h^31^. The mechanisms underlying the up-regulation of GFAT1 in pancreatic cancer may need further investigation.

Although the clinical significance of GFAT1 in pancreatic cancer has been presented in our study, known prognostic factors such as tumor grading, T-stage and M-stage fail to be significant in the univariable analysis. Some limitations of this study should be acknowledged. (1) The number of patients enrolled in this study was small. (2) Since pancreatic cancer are often diagnosed late when surgical resection is not applicable, the proportion of pancreatic cancer patients at advanced/late stage was relative low. (3) Single cohort seems to be inadequate to reach greater reliability. Thus, a large, multi-center, prospective data is needed to validate these results and more efforts need to be exerted in the future studies.

In conclusion, our study has identified aberrant expression of GFAT1 as an independent prognostic factor in our small number of pancreatic cancer patients, and GFAT1 expression could be integrated with pTNM stages to generate a nomogram to give a better risk stratification for pancreatic cancer patients with different prognosis. Future studies may focus on the mechanisms underlying the tumorigenic role of GFAT1 and the potential application of GFAT1 inhibitors in the treatment of pancreatic cancer.

## Materials and Methods

### Patients and specimens

For tissue microarray detection, tumor specimens including 96 pancreatic cancer tissues and 80 adjacent non-tumor tissues were obtained from patients who underwent surgical resection without preoperative treatment from 2004 to 2008, at Department of General Surgery, Zhongshan Hospital (Fudan University, Shanghai, P.R. China). The clinicopathological and baseline demographic characteristics of the patients, including age, gender, tumor size, tumor site, tumor differentiation, and tumor stage were retrospectively collected. Tumor stages were histologically classified according to the 7th Edition of the American Joint Committee On Cancer TNM classification. OS was calculated from the date of surgery to the date of death (or the last follow-up). Follow-up was terminated in December 2011. All methods were approved by the research medical ethics committee of Fudan University and were carried out in accordance with the approved guidelines. Informed consent on the use of clinical specimens were obtained from all patients.

### TCGA, GEO and ArrayExpress datasets

These data are publically available from the Cancer Genome Atlas and the GEO database (accession number: GSE3654^32^, GSE16515^33^, GSE28735^34^, GSE39751) and ArrayExpress database (E-MEXP-950^35^). For the TCGA dataset, all level-3 data were downloaded by using TCGA-Assembler software^36^. The mRNA expression in TCGA dataset was measured by RNA sequencing V2. The RSEM (RNA-Seq by Expectation-Maximization) counts were further normalized by TMM (trimmed mean of M value) method to estimate the relative RNA production levels using edgeR software^37^. For the GEO dataset and ArrayExpress database, the relative mRNA expression was achieved through the Oncomine database (https://www.oncomine.org/resource/login.html).

### Tissue microarray and immunohistochemistry

Tissue microarrays and immunohistochemistry analysis were performed as previously described^38^, and the tissue microarray was established with formalin-fixed paraffin-embedded surgical specimens. Primary anti-GFAT1 antibody (1:500; ab176775; Abcam, Cambridge, MA) was used for immunohistochemistry staining. The intensity of immunostaining was evaluated by two independent pathologists without the knowledge of clinicopathological data. Variations in the enumeration, within a range of 5%, were reevaluated, and a consensus decision was made. The staining intensity was sorted by 0 (negative), 1 (weak), 2 (moderate) and 3 (strong). Depending on the staining extent, the area was categorizes as 0 (<5%), 1 (5–25%), 2 (26–50%), 3 (51–75%) and 4 (>75%). To obtain an IHC score that takes into account the IHC signal intensity and the frequency of positive cells, we generated a composite expression scores (CES) with full range from 0 to 12^39^ (Supplementary Table S4).

### Statistical analysis

ROC analysis was conducted to select the optimum cut-off value of the staining score to dichotomize the patients into low and high groups^40^. Comparisons between GFAT1 expression and clinicopathologic variables were evaluated using chi-square test. Survival curves were conducted by Kaplan-Meier method and compared by log-rank test. The Cox proportional hazards regression model was applied to evaluate multivariate analyses, and those statistically significant characteristics in univariate analysis were used to perform multivariate analysis. Nomogram was set to construct the prognostic model using the Cox proportional hazards model which generates a hazard function h(t) (failure rate at time t for patients surviving to time t) as a function of the covariates^41^. The Cox proportional hazards model estimates the score of the TNM classification (0 for “IA”, 16 for “IB”, 33 for “IIA”, 49 for “IIB” or 82 for “IV”) and GFAT1 expression (0 for “Low” or 100 for “High”) for each patient. Calibration plot was used to evaluate the prognostic accuracy of the models. ROC analysis was conducted to compare the sensitivity and specificity for the prediction of OS by the prognostic models. Differences between two groups were tested with Student’s two-tailed t test. All statistical tests were two-tailed and differences were considered significant at level of <0.05. Data were analyzed using IBM SPSS Statistics 22.0 (SPSS, Chicago, IL, USA) and R software 3.2.2 with the “rms” package (R Foundation for Statistical Computing, Vienna, Austria).

## Additional Information

**How to cite this article**: Yang, C. *et al*. High expression of GFAT1 predicts poor prognosis in patients with pancreatic cancer. *Sci. Rep.* **6**, 39044; doi: 10.1038/srep39044 (2016).

**Publisher's note:** Springer Nature remains neutral with regard to jurisdictional claims in published maps and institutional affiliations.

## Supplementary Material

supplementary Information

## Figures and Tables

**Table 1:** Relationships between the expression level of GFAT1 and the clinicopathological variables of pancreatic cancer patients.

Variables	No.	GFAT1 expression		*P*-value
		Low No. (%)	High No. (%)	
Gender				0.218
Male	62	18 (29.0)	44 (71.0)	
Female	34	6 (17.6)	28 (82.4)	
Age (years)				0.724
>60	49	13 (26.5)	36 (73.5)	
≤60	47	11 (23.4)	36 (76.6)	
Tumor site				0.810
Head	58	14 (24.1)	44 (75.9)	
Body	38	10 (26.3)	28 (73.7)	
Tumor size (cm)				0.939
>3	66	17 (25.8)	49 (74.2)	
≤3	30	7 (25.0)	21 (75.0)	
G: tumor grading				0.355
I–II	85	23 (27.1)	62 (72.9)	
III–IV	11	1 (9.1)	10 (90.9)	
pT stage				1.000
pT1-pT2	76	19 (25.0)	57 (75.0)	
pT3-pT4	20	5 (25.0)	15 (75.0)	
pN stage				**0.020**
Absent	61	20 (32.8)	41 (67.2)	
Present	35	4 (11.4)	31 (88.6)	
pM stage				1.000
Absent	94	24 (25.5)	70 (74.5)	
Present	2	0 (00.0)	2 (100.0)	
pTNM stage				**0.033**
I	42	15 (35.7)	27 (64.3)	
II–IV	54	9 (16.7)	45 (83.3)	

Abbreviations: *P*-value was got by pearson chi-square tests. *P* < 0.05 indicated the differences had statistical significance.

**Table 2:** Univariate Cox regression analysis of clinicopathological characteristics influencing the overall survival of pancreatic cancer patients.

Variables	Univariate		
	HR	95% CI	*P*-value
Gender			0.265
Male vs female	1.337	0.803–2.226	
Age (years)			0.366
>60 vs ≤60	1.257	0.765–2.064	
Tumor site			0.490
Body vs head	1.195	0.721–1.980	
Tumor size (cm)			0.715
≤3 vs >3	1.103	0.650–1.872	
G: tumor grading			0.196
III–IV vs I–II	1.766	0.747–4.175	
pT stage			0.802
pT1-pT2 vs pT3-pT4	1.081	0.587–1.990	
pN stage			**0.004**
Present vs absent	2.248	1.300–3.887	
pM stage			0.357
Present vs absent	2.513	0.353–17.88	
pTNM stage			**0.002**
II–IV vs I	2.170	1.319–3.571	
GFAT1 expression			**<0.001**
High vs low	3.126	1.856–5.263	

Abbreviations: CI, confidence interval; HR, hazard ratio; *P* < 0.05 was considered statistically significant.
