# Supplementary material for: High expression of GFAT1 predicts poor prognosis in patients with pancreatic cancer
Source: Sci Rep. 2016 Dec 20;6:39044. doi: 10.1038/srep39044 (PMC5172351; doi:10.1038/srep39044)
Supplement: supplementary Information [file srep39044-s1.pdf]

# High expression of GFAT1 predicts poor prognosis in patients with pancreatic cancer

Caiting Yang[1,2], Peike Peng[1,2], Lili Li[1,2], Miaomiao Shao[1,2], Junjie Zhao[3], Lan Wang[1,2], Fangfang Duan[1,2,4], Shushu Song[1,2], Hao Wu[1,2], Jie Zhang[2,4], Ran Zhao[4], Dongwei Jia[1,2], Mingming Zhang[1,2], Weicheng Wu[1,2], Can Li[1,2], Yefei Rong[3], Lei Zhang[3,*], Yuanyuan Ruan[1,2,*], Jianxin Gu[1,2,4]

**Table S1. The IHC CES score for GFAT1 low and high expression**

| Classification | Score | No. | Percent (%) |
|---|---|---|---|
| Low | 1 | 1 | 1.04 |
| | 2 | 1 | 1.04 |
| | 3 | 4 | 4.17 |
| | 4 | 8 | 8.33 |
| | 5 | 1 | 1.04 |
| | 6 | 6 | 6.25 |
| High | 7 | 13 | 13.54 |
| | 8 | 39 | 40.63 |
| | 9 | 0 | 0 |
| | 10 | 0 | 0 |
| | 11 | 4 | 4.17 |
| | 12 | 16 | 16.67 |

**Table S2. The IHC intensity score for GFAT1 expression**

| Classification | Score | No. | Percent (%) |
|---|---|---|---|
| Negative | 0 | 2 | 2.08 |
| Weak | 1 | 15 | 15.63 |
| Moderate | 2 | 59 | 61.46 |
| Strong | 3 | 20 | 20.83 |

**Table S3. The IHC frequency score for GFAT1 expression**

| Classification | Score | No. | Percent (%) |
|---|---|---|---|
| <5% | 0 | 3 | 3.13 |
| 5%-25% | 1 | 2 | 2.08 |
| 26%-50% | 2 | 7 | 7.29 |
| 51%-75% | 3 | 21 | 21.88 |
| >75% | 4 | 63 | 65.63 |

**Table S4. Calculation of the IHC composite expression score**

| Intensity score | Frequency score | | | |
|---|---|---|---|---|
| | **1 (5%-25%)** | **2 (26%-50%)** | **3 (51%-75%)** | **4 (76%-100%)** |
| **0 (negative)** | 0 | 0 | 0 | 0 |
| **1 (weak)** | 1 | 2 | 3 | 4 |
| **2 (moderate)** | 5 | 6 | 7 | 8 |
| **3 (strong)** | 9 | 10 | 11 | 12 |

Composite expression score (CES) is calculated from intensity and frequency measurements for immunostaining. CES = 4(intensity score - 1) + frequency score

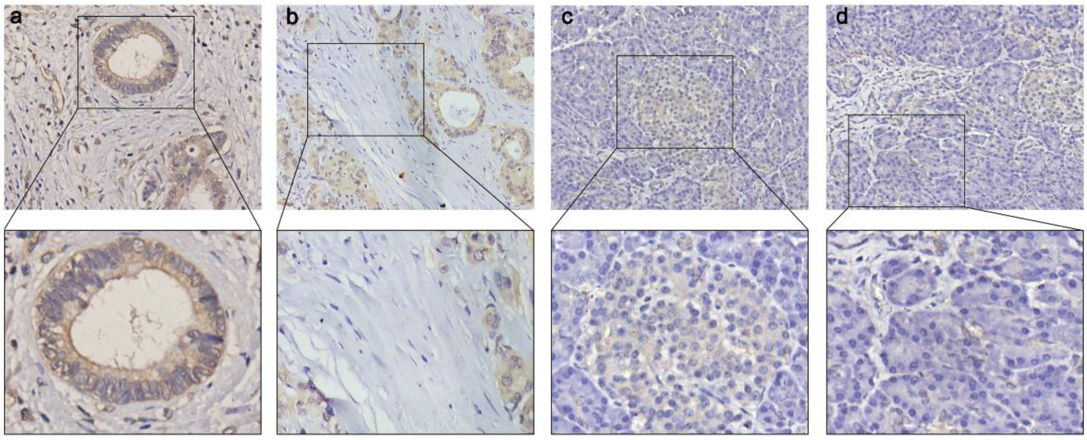


**Supplementary Figure S1. GFAT1 expression in different cellular compartments of pancreatic cancer tissues.** Representative IHC staining images of GFAT1 and its regional magnification are shown. **(a)** Epithelial tumor cells. **(b)** Stroma. **(c)** Islets. **(d)** Acinar cells. Original magnification: 200×.

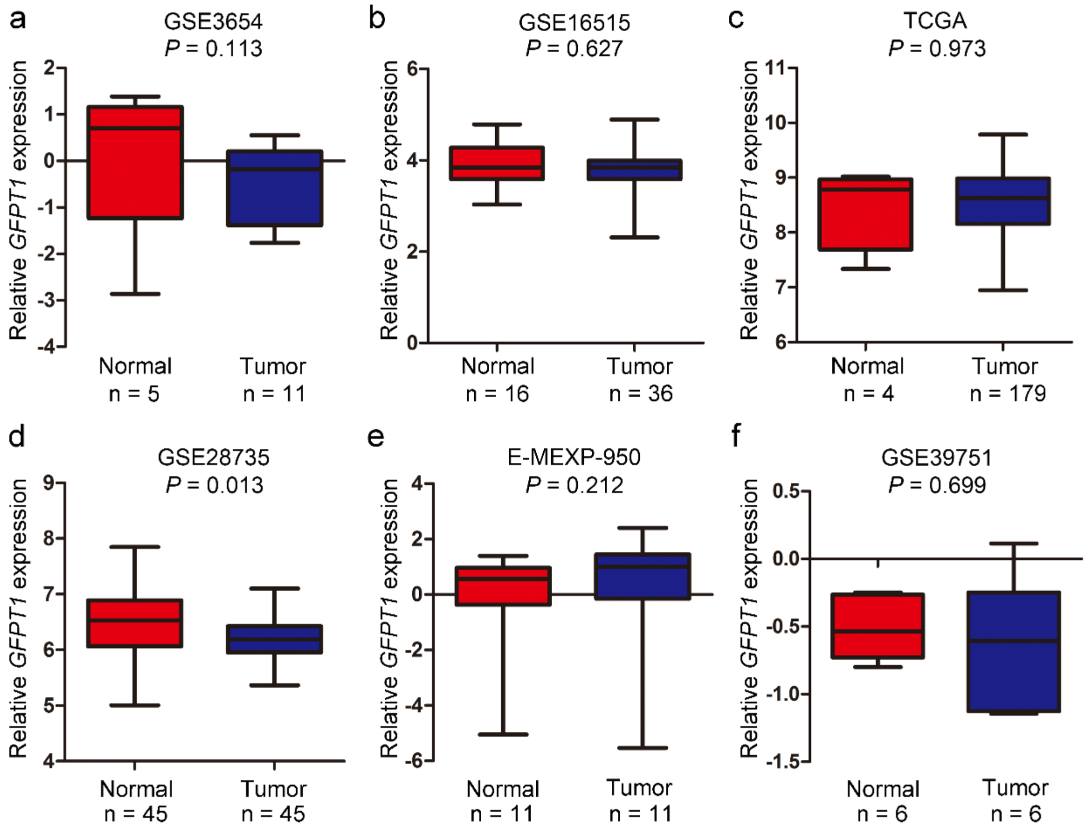


**Supplementary Figure S2. The expression patterns of phosphoacetylglucosamine mutase in pancreatic cancer tissues.** Relative expression of phosphoacetylglucosamine mutase mRNA (*pgm3*) in pancreatic cancer and normal pancreatic tissues in GSE3654 **(a)**, GSE16515 **(b)**, TCGA datasets **(c)**, GSE28735 **(d)**, E-MEXP-950 **(e)** and GSE39751 **(f)** datasets.

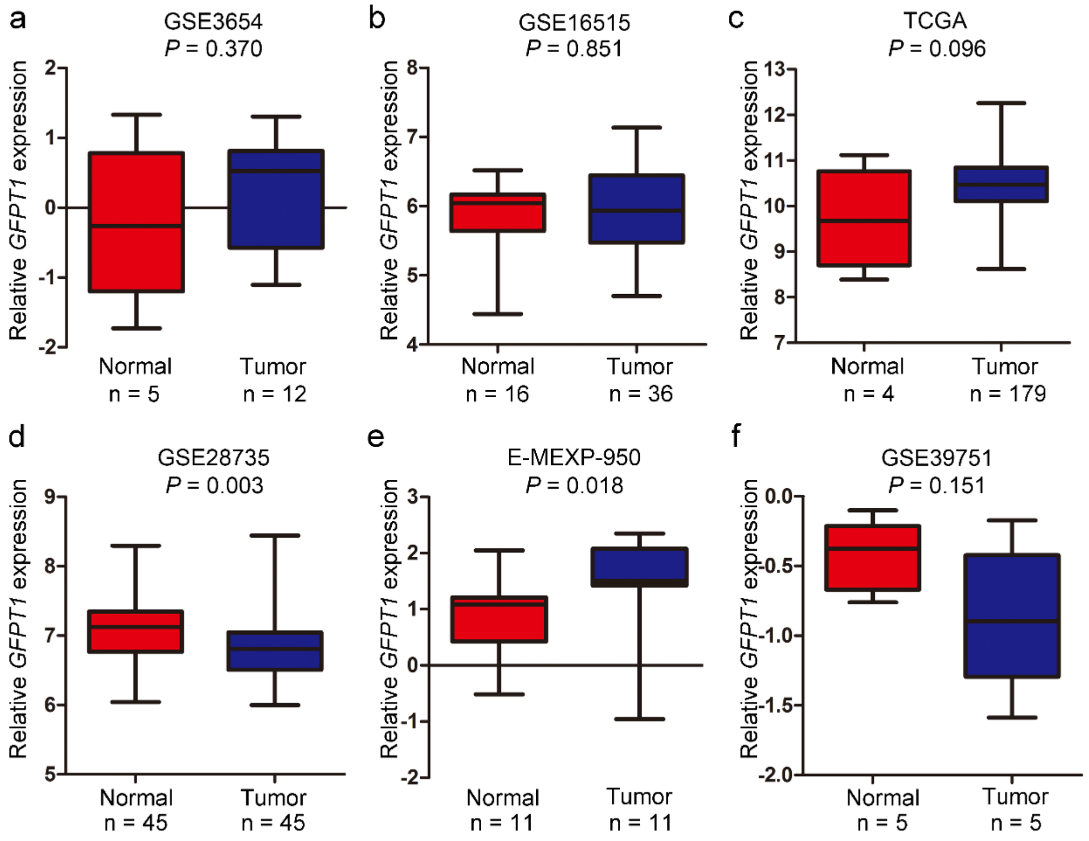


**Supplementary Figure S3. The expression patterns of UDP-N-acetylglucosamine pyrophosphorylase in pancreatic cancer tissues.** Relative expression of UDP-N-acetylglucosamine pyrophosphorylase mRNA (*uap1*) in pancreatic cancer and normal pancreatic tissues in GSE3654 **(a)**, GSE16515 **(b)**, TCGA datasets **(c)**, GSE28735 **(d)**, E-MEXP-950 **(e)** and GSE39751 **(f)** datasets.

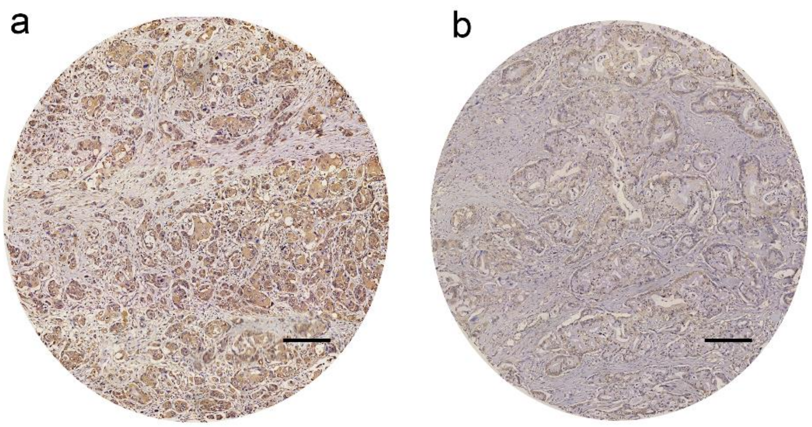


**Supplementary Figure S4. Representative images of GFAT1 expression in pancreatic cancer tissue. (a)** Tumor tissue with GFAT1 high staining. **(b)** Tumor tissue with GFAT1 low staining. Scale bar = 200 μm.
